# The antioxidant level of Alaska’s wild berries: high, higher and highest

**DOI:** 10.3402/ijch.v72i0.21188

**Published:** 2013-08-05

**Authors:** Roxie Rodgers Dinstel, Julie Cascio, Sonja Koukel

**Affiliations:** 1 University of Alaska Fairbanks, Fairbanks, AL, USA; 2 New Mexico State University, Las Cruces, NM, USA

**Keywords:** antioxidants, berries, nutritional value, Alaska, phytochemical

## Abstract

**Background:**

In the last few years, antioxidants have become the stars of the nutritional world. Antioxidants are important in terms of their ability to protect against oxidative cell damage that can lead to conditions, such as Alzheimer’s disease, cancer and heart disease – conditions also linked with chronic inflammation. The antioxidant and anti-inflammatory effects of Alaska’s wild berries may have the potential to help prevent these diseases.

**Objective:**

To discover the antioxidant levels of Alaska wild berries and the ways these antioxidant levels translate when preservation methods are applied to the berry.

**Design:**

This research centred on both the raw berries and products made from the berries. In the first year, a variety of wild berries were tested to discover their oxygen radical absorption capacity (ORAC) in the raw berries. The second level of the research project processed 4 different berries – blueberries, lingonberries, salmonberries, highbush cranberries – into 8 or 9 products made from these berries. The products were tested for both ORAC as well as specific antioxidants.

**Results:**

The Alaska wild berries collected and tested in the first experiment ranged from 3 to 5 times higher in ORAC value than cultivated berries from the lower 48 states. For instance, cultivated blueberries have an ORAC scale of 30. Alaska wild dwarf blueberries measure 85. This is also higher than lower 48 wild blueberries, which had a score of 61. All of the Alaskan berries tested have a level of antioxidant considered nutritionally valuable, ranging from 19 for watermelon berries to 206 for lingonberries on the ORAC scale. With the processed products made from 4 Alaska wild berries, one of the unexpected outcomes of the research was that the berries continued to have levels of antioxidants considered high, despite the effects of commonly used heat-processing techniques. When berries were dehydrated, per gram ORAC values increased.

**Conclusion:**

Alaska wild berries have extraordinarily high antioxidant levels. Though cooking lowered the antioxidant level, and adding ingredients such as sugar diluted the antioxidant concentration, products made from berries are high sources of antioxidants.

Consumption of fruits and vegetables has been correlated to robust improvement in brain health, cancer prevention and heart health. The US Department of Health and Human Services in partnership with the US Department of Agriculture (1) published the *Healthy People 2010* report with recommendations that all people over 2 years of age increase their fruit consumption to a minimum of 2 fruit servings per day. Currently, the Centers for Disease Control and Prevention (2) reports that only 33% of adults meet this recommended daily allowance. The consumption of fruits, particularly berries, and their ability to promote health is a subject receiving a great deal of attention. Scientists around the world are investigating ways in which the natural compounds found in fruits and wild berries may help combat diseases and promote healthy aging.

Epidemiological studies (3) have reported an association between dietary increase in fruit and vegetable intake with a decrease of cardiovascular disease and the breakdown of neural tissue. Berries and leafy vegetables were found to provide the greatest health benefits. Researchers Heim, Angers, Léonhart and Ritz (4) attributed the health benefits to phenolic constituents and antioxidant activity.

According to research conducted by Michenaud-Rague et al. (5), wild blueberries (*Vaccinium angustifolium*) are rich sources of polyphenols (e.g. flavonols, phenolic acids, anthocyanins) found to decrease the risk of cardiovascular and degenerative diseases. Furthermore, Riso et al. (6) investigated the effect of regular consumption of a wild blueberry or a placebo drink on markers of oxidative stress, inflammation and endothelial function in subjects with risk factors for cardiovascular disease. Results indicated the consumption of the wild berry drink for 6 weeks significantly reduced the levels of oxidized DNA bases and increased the resistance to oxidatively induced DNA damage.

A 2012 study of more than 36,000 women was conducted to examine the relationship between dietary antioxidant levels and strokes. Rautiainen and Larsson (7) concluded that “dietary TAC (Total Antioxidant Capacity) is inversely associated with total stroke among cardiovascular disease-free [CVD] women and hemorrhagic stroke among women with a CVD history” (p. 335). The oxygen radical absorbance capacity (ORAC) was used to measure the antioxidant level.

According to studies conducted by Prior et al. (8), wild blueberries have the highest antioxidant capacity of 20 fruits tested for ORAC. Furthermore, a research team concluded that 1 cup of wild blueberries had more ORAC value than strawberries, plums, raspberries, or cultivated blueberries (9). Prior et al. (10) drew these conclusions from an antioxidant capacity study:

In general, blueberries are one of the richest sources of antioxidant phytonutrients of the fresh fruits and vegetables we have studied. The polyphenolic components present within blueberries may have multiple health benefits which at this point are difficult to understand. (p. 2686 and 2692, respectively).

A 2007 study (11) determined that antioxidant levels in the body can be altered by diet, but the absorption of these antioxidants depends on the level of antioxidants, the adsorption/metabolism and the amount consumed. The research team recommended that individuals consume high antioxidant foods with each meal to prevent oxidative stress that occurs after a meal.

Oxidative stress is an important factor in ischemic stroke – a stroke occurring as a result of an obstruction with a blood vessel supplying blood to the brain (12). Results from a study conducted by Sweeney et al. (13) found that adding blueberries to the diet of rats can reduce by half the effects of ischemic stroke.

Joseph et al. (14) studied the effect of a diet high in fruit and vegetable extracts on rats. The team determined that a diet supplemented with extracts from strawberries, blueberries, or spinach improved motor skills and short-term memory loss. Further studies (15) suggested that berry supplementation could overcome the genetic predisposition to Alzheimer’s disease.

The anti-inflammatory potential of the polyphenols in blueberries, including the potent antioxidant anthocyanin, was the focus of a 2008 study (16). When rats with neuronal lesions were fed a blueberry-supplemented diet, not only did they perform better in cognitive tests, the concentration of several substances in the brain that can trigger an inflammatory response was significantly reduced. The polyphenols in blueberries appear to inhibit the production of these inflammatory mediators.

The first research on humans (17) was conducted at the University of Cincinnati. A diet supplemented by wild blueberries was shown to improve memory function and mood in older adults with memory decline. The findings suggested that regular consumption of wild blueberries may slow cognitive function declines and decrease depression in older adults.

Researchers from Brigham and Woman’s Hospital in Boston (18) investigated whether a diet rich in berries would slow cognitive decline. Over a 20-year period, the Nurses’ Health Study evaluated 16,000 participants regarding cognitive decline and consumption of berries. Results indicated that greater intakes of strawberries and blueberries were associated with slower cognitive decline. The researchers determined that higher intakes of flavonoids, particularly from berries, appeared to reduce rates of cognitive decline in older adults.

Though berries have been shown to have a positive effect on cognitive function and age-related disorders, it remains unclear as to how this happens. Poulose and Joseph (19) suggested that the damage caused by aging is precipitated by a reduction in the brain’s natural cleaning processes. The researchers contend that as individual’s age, the brain’s “housekeeping” mechanisms fail, allowing the build-up of toxic proteins that contribute to mental decline. The body cannot protect itself from inflammation and oxidative damage. Polyphenolics found in fruits, vegetables and nuts have antioxidant and anti-inflammatory properties that can protect against age-related decline. Research results indicated a reversal of age-related problems in nerve functions and behaviors that involved cognition and memory when aged laboratory rats were given strawberry, blueberry or blackberry extracts.

The nutritional value of berries is well documented (20). However, the antioxidant levels of berries were largely unknown before researchers (14) presented results that indicated commercial berries and fruits showed a high level of antioxidants. The purpose of this research was to evaluate antioxidant levels of selected Alaska wild berries.

Berries are small fleshy fruits low in calories, high in moisture and contain both soluble and insoluble fibres (20). The fruits contain natural antioxidants, such as vitamins C and E, low levels of sodium and large amounts of potassium. They are naturally rich in micronutrients: folic acid, calcium, selenium, α- and β-carotene, and lutein. Phytochemicals present in berries are polyphenols, flavonoids, anthocyanins and ellagitannins.

Berry picking is a cherished tradition among all Alaskans that provides important physical and recreational activities for young and old alike. It has been an integral part of subsistence activities for thousands of years. Alaska is rich in wild edible berries that provide essential nutrients, especially vitamin C, and antioxidants for northern climates.

As previously mentioned, a method of measuring antioxidant capacity of foods is through an ORAC test. The ORAC scale compares items among food groups to show relative antioxidant activity. ORAC is a measure of water-soluble antioxidant levels and does not distinguish among those antioxidants that have benefits to humans and those that do not. It is simply an overall estimate of antioxidant activity. *Note:* Due to the recent controversy, the USDA’s Nutrient Data Laboratory (NDL) removed the ORAC database from the NDL website (21).

The 1999 (14) research results found cultivated blueberries received the highest ORAC levels of commercial fruits – a score of about 20 (scores above 40 are considered very high). Holloway et al. (15) designed a similar study using Alaska wild berries. Laboratory tests showed that Alaska wild berries are a rich source of antioxidants. On the ORAC test, almost all Alaskan wild berries tested scored greater than 20. Figure 1 provides the ORAC score for the berries.

**Fig. 1:**
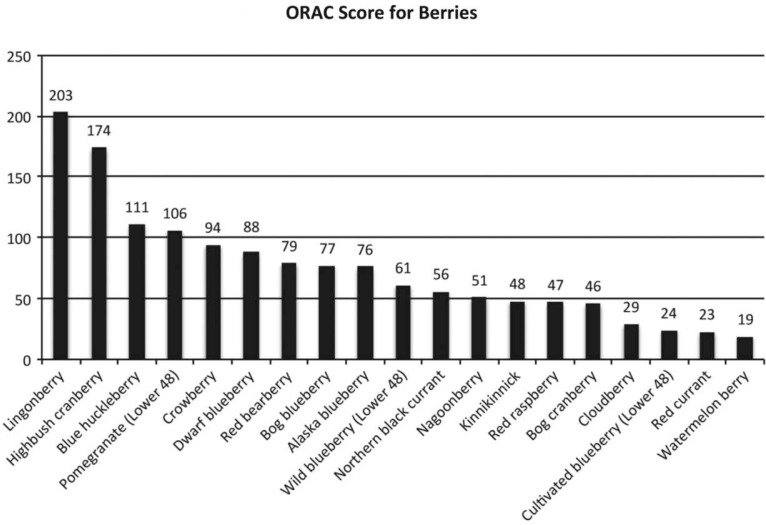
ORAC scores of Alaska’s native wildberries.

Though Alaskans eat fresh berries, the bulk of the collected berries are home-processed into products that are used throughout the year. Thereby, additional research was conducted to test the antioxidant levels on home-processed berry products. Using recommended recipes from Alaska Cooperative Extension, a variety of processed products were made from each of 4 types of wild berries: lingonberry (*Vaccinium vitis-idaea supsp. minus*), bog blueberry (*Vaccinium uliginosum*), salmonberry (*Rubus spectabilis*) and highbush cranberry (*Viburnum edule*).

## Procedures

Thirty gallons of fruit were harvested from a variety of locations throughout Alaska. For each type of berry, all collected samples were combined and thoroughly mixed to form a single berry sample. Berries were frozen and held at −10°F to wait processing. The frozen berries were then divided at random into 3 replicates. Sufficient berries to make each recipe were taken at random from each replicate and were processed into the following products:

- Jam
- Juice (canned and frozen)
- Syrup
- Sauce
- Fruit leather
- Dried fruit
- Freezer jam

Originally, 9 products were to be made from each type of berry. In practice, however, some of the berries were unsuitable in certain products, so these products were omitted leaving the 8 products as listed above.

Three identical batches of each product were prepared, one from each replicate of berries, all completed within 1 week. Recipes were prepared using standard equipment and processes accepted in home food preservation. A household freezer, a water bath canner, home dehydrator, juicer and home-style range were used in preparation of the products.

The processed berry products were shipped overnight from Fairbanks, Alaska to Brunswick Laboratories in Wareham, Massachusetts. These laboratories were chosen because this is where the original laboratory testing for the 1999 (14) research was performed.

The Brunswick laboratories analyzed the berry samples for H-ORAC using the scavenging capacity of the peroxyl radical against a fluorescent probe (9, 22). The levels of quercetin were determined on high-performance liquid chromatography after extraction in ethyl acetate and reconstitution in methanol at Brunswick Laboratories. Data were analyzed by analysis of variance for randomized block design with blocks being different processing dates for each replicate.

**Fig. 2:**
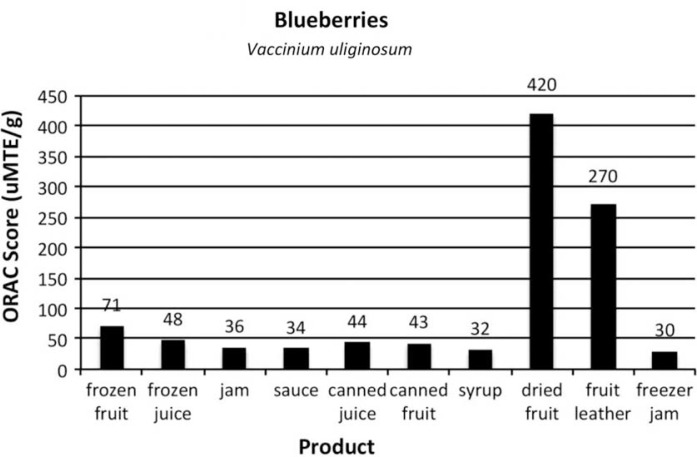
ORAC scores of items commonly prepared from Alaska blueberries, *Vaccinium uliginosum*.

**Fig. 3:**
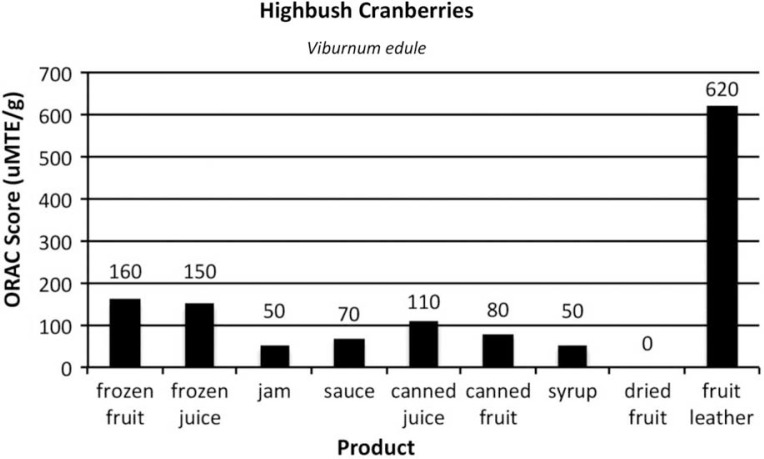
ORAC scores of items commonly prepared from high bush cranberries, *Viburnum edule*.

**Fig. 4:**
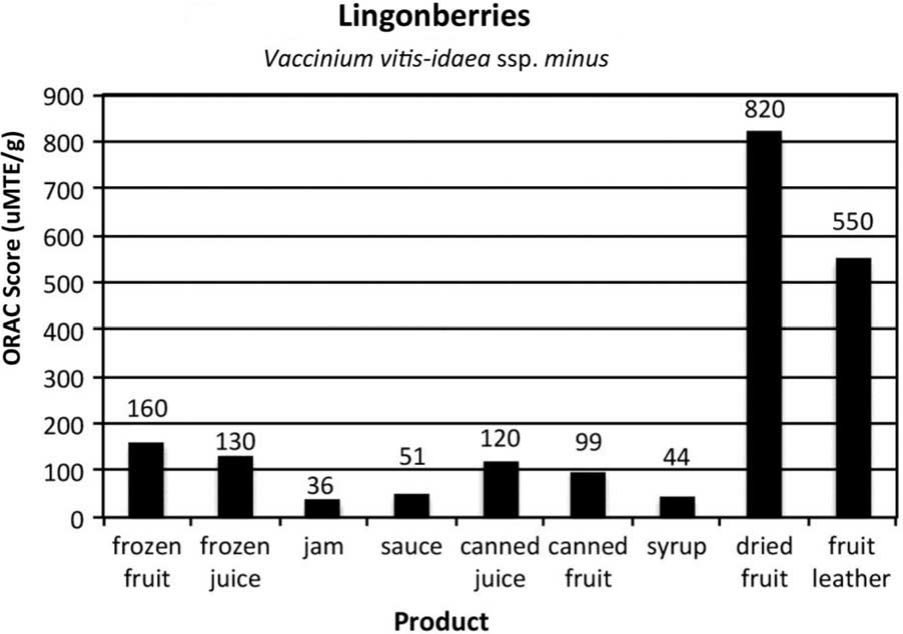
ORAC scores of items commonly prepared from low bush cranberries, *Vaccinium vitis-idaea* ssp.*minus*.

**Fig. 5:**
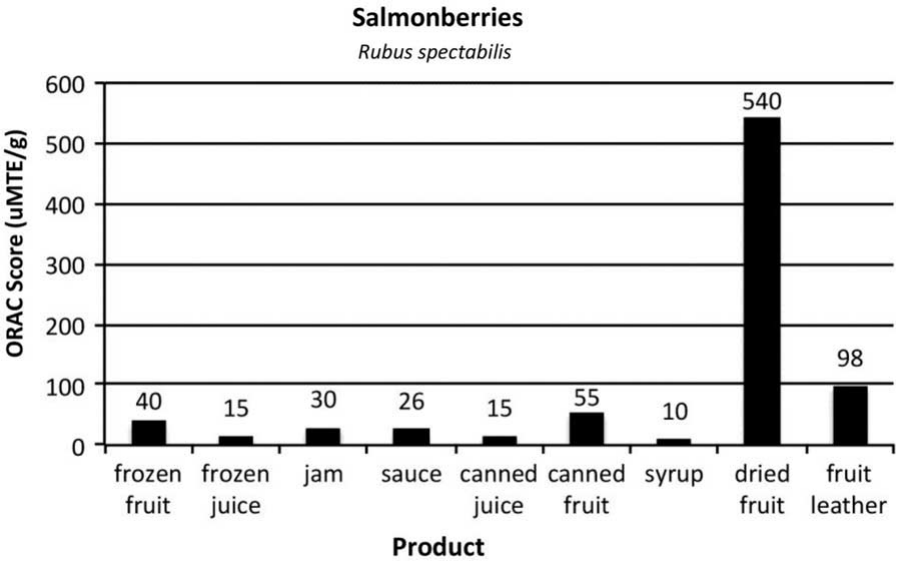
ORAC scores of items commonly prepared from salmonberries, *Rubus spectabilis*.

Figures 2–5 provide the ORAC values for the product samples analyzed for each of the 4 types of berries.

The ORAC value of the frozen control for the blueberries was slightly lower than the value of the same berry type in the 2004 experiment. The berries were gathered in different years, which may account for the difference. Additionally, the frozen berries were held in the freezer longer to allow for the products to be made.

Highbush cranberry analyses showed the juice to be almost as high in ORAC value as the frozen berry control, despite crushing the berry in a juicer to get the juice, and removing pulp and seeds. Because of their flavour and large flat stone, highbush cranberries are not suitable for drying whole; therefore whole dried berries were not tested. The puree, seeds removed, was used to make fruit leather.

The ORAC value of the frozen control for the lingonberries was lower than the value of the same berry type in the 2004 experiment. The berries were gathered in different years, which may account for the difference. Additionally, the frozen berries were held in the freezer longer to allow for the products to be made.

Salmonberries were not collected in the 2004 wild berry study, but were gathered and made into products for the berry product study.

## Discussion

The Alaska wild berries collected and tested in the first experiment ranged from 3 to 5 times higher in ORAC value than their cultivated cousins. For instance, cultivated blueberries have an ORAC scale of 30. Alaska Blueberry (*Vaccinium alaskensis*) measured 76. All wild blueberry varieties in Alaska – Blue Huckleberry (*Vaccinium ovalifolium*) 111, dwarf blueberries (*Vaccinium caespitosum*) 85, Bog Blueberry (*Vaccinium uliginosum*) 77 – scored higher than wild blueberries from the contiguous 48 states, which measured 61.

All of Alaska’s berries that were tested in the first experiment have a level of antioxidant considered nutritionally valuable, ranging from 19 for watermelon berries to 206 for lingonberries on the ORAC scale. Cloudberries, red currants and watermelon berries (antioxidant levels 19,23 and 29, respectively) are on the low end of the ORAC scale for the Alaska wild berries tested. Bog cranberry (*Oxycoccux microcarpus*), red raspberry (*Ribes triste Pallas*.), kinnikinnick (*Arctostaphylos uva-ursi*), Nagoonberry (*Rubus arcticus*) and Northern Black Currant (*Ribes hudsonian Rich*.) scored from 45 to 56.

With the processed products made from 4 Alaska wild berries, one of the unexpected outcomes of the research was that the berries continued to have high levels of antioxidants despite the effects of commonly used heat-processing techniques. ORAC values in canned berries decreased by one-third (in blueberries and lingonberries) and by one-half in highbush cranberries. Despite this decrease, the ORAC values were still high compared to other fruits. It is postulated that the increased ORAC score of the canned salmonberries per gram is due to the structure of the berry: more fruit was compacted in a 1 g sample after being heated and canned than in the frozen control.

Other forms of heat-treated products also had sugar added. The sugar added weight to the products, so when taking a gram sample, the berry was “diluted” by the sugar and pectin (when used) in the sauce, jam and syrup. Each berry, and product, varies in the amount of sugar added.

For juice, the berries were run through a juicer machine to remove seeds and pulp. It is interesting to note that between the frozen juice and the canned juice, which was heat-processed 5 min in a boiling water canner, the ORAC value decreased only slightly. In blueberries, frozen juice was 48, while canned juice was 43. For lingonberries, frozen juice was 130 and canned juice was 120. Highbush cranberry juice seemed the most affected by the heat processing as frozen juice scored 150, while canned juice scored 110. Salmonberry juice had the same ORAC value in frozen and canned form.

When berries were dehydrated, per gram ORAC values skyrocketed. Dried lingonberries had an ORAC score 5 times higher than the control. Lingonberry fruit leather was 3.4 times higher than the control. Blueberry and highbush cranberry fruit leathers were 3.8 times higher than their respective controls. Drying fruits and making fruit leather concentrated the skin and pulp, and removed moisture, significantly increasing the antioxidant levels in each gram of product. While drying whole berries can take days, making berry puree into fruit leather takes a third of the time and is a practical method for enjoying the concentrated antioxidant benefits of berries out of season.

When comparing ORAC data, care must be taken to ensure the units and foods being compared are similar. The typical quantity of berry or berry product that will be eaten should be considered; jam may have significant ORAC, but smaller quantities are eaten at a time than fresh berries. Similarly, dried berries or fruit leather are very high in ORAC, but also have concentrated amounts of sugar so should be consumed with moderation. Also, ORAC gives the overall antioxidant capacity of a food. It does not specify what kinds of antioxidants are present.

## Conclusions

The research results indicated that most home processing methods reduced antioxidant levels in products using Alaska wild berries. However, the product ORAC scores remained very high compared to other fruit that had been tested in previous research. Testing revealed that dried fruits and fruit leathers concentrated the skin and pulp and removed moisture, thereby significantly increasing the antioxidant levels in each gram of product. The results of this study reaffirm that Alaska wild berries are a great source of nutrients. Home processing methods do not eliminate the naturally occurring antioxidants and fruit drying methods concentrate the antioxidants to extremely high levels. Gathering berries, then processing them for longer storage, provides healthy food in addition to benefits of physical activity, fresh air, spending time with family and friends and engaging in traditional and subsistence activities.
